# Psychological distress in men with prostate cancer undertaking androgen deprivation therapy: modifying effects of exercise from a year-long randomized controlled trial

**DOI:** 10.1038/s41391-021-00327-2

**Published:** 2021-02-08

**Authors:** Daniel A. Galvão, Robert U. Newton, Suzanne K. Chambers, Nigel Spry, David Joseph, Robert A. Gardiner, Ciaran M. Fairman, Dennis R. Taaffe

**Affiliations:** 1 Exercise Medicine Research Institute, Edith Cowan University, Joondalup, WA Australia; 2 School of Medical and Health Sciences, Edith Cowan University, Joondalup, WA Australia; 3 Faculty of Health, University of Technology Sydney, Ultimo, NSW Australia; 4 Faculty of Medicine, University of Western Australia, Nedlands, WA Australia; 5 Department of Radiation Oncology, Sir Charles Gairdner Hospital, Nedlands, WA Australia; 6 Department of Urology, Royal Brisbane and Women’s Hospital, Brisbane, QLD Australia

**Keywords:** Diseases, Urological cancer

## Abstract

**Objectives:**

To assess the long-term effects of various exercise modes on psychological distress in men with prostate cancer on androgen deprivation therapy (ADT).

**Patients and methods:**

135 prostate cancer patients aged 43–90 years on ADT were randomized to twice weekly supervised impact loading and resistance exercise (ImpRes), supervised aerobic and resistance exercise (AerRes), and usual care/delayed supervised aerobic exercise (DelAer) for 12 months, and completed measures of psychological distress using the Brief Symptom Inventory-18 (BSI-18). BSI-18 provides three subscales for anxiety, depression, and somatisation, as well as the global severity index (GSI) where higher scores indicate higher distress.

**Results:**

Following the intervention, somatization was not different to baseline, however, there were significant interactions (*p* < 0.01) for depression, anxiety, and the GSI. In ImpRes, depression was reduced at 12 months compared to baseline and 6 months (0.78 ± 1.39 vs. 1.88 ± 3.24 and 1.48 ± 2.65, *p* < 0.001), as was the GSI (3.67 ± 4.34 vs. 5.94 ± 7.46 and 4.64 ± 4.73, *p* < 0.001) with anxiety reduced compared to baseline (1.08 ± 1.54 vs. 1.98 ± 2.56). Depression and the GSI decreased (*p* < 0.05) in AerRes at 6 months but increased by 12 months, while in DelAer the GSI was reduced at 12 months compared to 6 months (3.78 ± 3.94 vs. 5.25 ± 4.22, *p* = 0.031). Men with the highest level of anxiety, depression, somatization, and the GSI improved the most with exercise (*p*_trend_ < 0.001).

**Conclusion:**

Various supervised exercise modes (aerobic, resistance and impact loading) are effective in reducing psychological distress in men with prostate cancer on ADT. Those with the highest level of psychological distress improved the most. Supervised exercise should be prescribed to improve psychological health in prostate cancer patients on ADT.

## Introduction

Androgen deprivation therapy (ADT) is a common treatment for men with prostate cancer (PCa) [1]. Whilst effective for cancer control, ADT is associated with a myriad of physiological adverse effects, including osteoporosis, reduced muscle mass and strength, and increased adiposity [2–4]. Importantly, the psychological impact of the disease and its treatment remains a considerable burden, with numerous reports documenting significant psychological distress in men with PCa undergoing ADT [3–6]. Moreover, emerging evidence indicates that ADT may be associated with greater psychological distress compared to other PCa treatments. For example, ADT has been associated with a threefold greater risk of depression compared to radiation alone in men treated for recurrent PCa [6]. In addition, longer ADT duration has been associated with an increased risk of depression and inpatient and outpatient psychiatric treatments in men with localized PCa [7]. The impact of psychological distress in men with PCa, particularly when not addressed, can result in compromised quality of life [5, 8]. Importantly, men with PCa and with depressive disorders are less likely to undergo definitive therapy and are at increased risk of suicide [9–11]. Consequently, the management of psychological distress in patients with PCa should be an important clinical objective in efforts to improve quality of life and clinical outcomes [12, 13].

We have previously observed that physically inactive men with PCa experience higher global distress and anxiety than those who were physically active [14]. In non-PCa populations, exercise has been shown to have a significant antidepressant effect in people with depression [15], and in those with other chronic conditions [16]. Recently, exercise interventions that included specifically resistance-based exercise have been shown to reduce depressive symptoms in adults [17]. Nevertheless, in our previous systematic review on interventions to improve PCa survivorship, we reported that there were insufficient data available to determine the effects of exercise on depression or anxiety outcomes [18]. Similarly, others have only reported initial positive trends for the effect of exercise on anxiety and depression in men with PCa with few studies available [19]. Therefore, despite increasing evidence in other clinical populations, there is limited research examining the impact of exercise and different modes of exercise on psychological distress in men with PCa. In this report, we examined the effects of various exercise modes on psychological distress in men with PCa on ADT who undertook a year-long randomized controlled trial. We hypothesized that a year-long intervention of different exercise modes would result in reduced psychological distress and improved physical function in men with PCa on ADT.

## Patients and methods

Two hundred and ninety-three patients with PCa were screened for participation from 2009 to September 2012 in Perth, Western Australia and Brisbane, Queensland and their progress through the study has been previously described [20]. In brief, 130 patients were excluded for various reasons which included: declined to participate, too far to travel, ineligible, and unable to obtain physician consent resulting in 163 patients entering the study. The Brief Symptom Inventory-18 (BSI-18) to assess psychological distress was included after recruitment commenced and was administered to 135 patients and this forms the basis for this report. Inclusion criteria included: histologically documented PCa, a minimum exposure to ADT of 2 months, without prostate specific antigen (PSA) evidence of disease activity, and anticipated to remain hypogonadal for the subsequent 12 months. Regarding without PSA evidence of disease activity, this was operationalized in two ways by the study clinicians (NS, DJ). For those who recently initiated ADT, PSA needed to fall significantly and anticipated to remain low for the next year, while for those with established disease, low PSA had to remain low and stable with no indication of relapse in the next year. Exclusion criteria included: bone metastases, musculoskeletal, cardiovascular, or neurological disorders that could inhibit them from exercising as determined by their physician, inability to walk 400 m or undertake exercise, and resistance training performed in the previous 3 months. All participants obtained medical clearance from their physician. The study was approved by the University Human Research Ethics Committee and all participants provided written informed consent.

### Study design

This was a three-armed RCT with the primary endpoints being bone mineral density and cardiovascular capacity which we have reported previously [21, 22], and secondary endpoints including self-reported patient outcomes and physical function. Of the secondary outcomes, we have reported on fatigue [20]. As previously reported [20–22], potential participants were identified by their treating urologist/oncologist and referred to the study coordinator. Following a familiarization session and baseline assessment, the 135 participants in this report were randomly allocated to either: impact loading + resistance training (ImpRes, *n* = 49), aerobic + resistance training (AerRes, *n* = 50), or to usual care/delayed exercise (DelAer, *n* = 36) stratified according to time on ADT (< or ≥ 6 months). During the initial 6-month period 12 patients in ImpRes and 6 in AerRes discontinued the intervention, and 9 in DelAer were lost to follow-up. During the second 6-month period, three, ten, and three patients from ImpRes, AerRes, and DelAer, respectively, ceased participation. The main reasons for discontinuing were no longer interested in participating, poor health, injury, and moved away or no longer contactable.

### Exercise program

The exercise program has been previously described in detail [20, 21, 23]. In brief, ImpRes underwent 12 months of supervised exercise twice weekly in University-affiliated exercise clinics. The impact-loading component to target bone consisted of a series of bounding, skipping, drop jumping, hopping, and leaping activities that were progressive in nature. Resistance training consisted of six principal exercises as well as supplementary exercises that targeted the major upper and lower body muscle groups with 2–4 sets of each exercise performed at an intensity of 6–12 RM (maximal weight that can be lifted 6–12 times). In addition, the ImpRes group undertook training at home 2 days/week that consisted of skipping, hopping, leaping, and drop jumping. The AerRes group underwent supervised exercise in the clinic twice weekly for the initial 6 months consisting of 20–30 min of aerobic-based exercise such as walking/jogging and cycling or rowing on stationary ergometers at 60–85% of estimated maximal heart rate (HR), and the same resistance exercise program undertaken by ImpRes. In addition, participants were encouraged to undertake home-based aerobic activity such as walking/cycling with the goal to accumulate 150 min/week of aerobic-based activity. During the second 6-month period, patients were provided with a home-based maintenance program which required 150 min/week of aerobic activity and resistance exercise using body weight and elastic bands. The DelAer group were provided with a printed booklet with information about exercise for the initial 6 months, followed by 6 months of twice weekly exercise on a cycle ergometer at an intensity of ~70% HR max for up to 30–40 min and flexibility exercises in the clinic under supervision. All supervised exercise sessions for ImpRes, AerRes, and DelAer were undertaken with the guidance of an Accredited Exercise Physiologist and included small groups of up to ten participants. During the 12-month study period, all participants were asked to maintain customary physical activity and dietary patterns.

### Psychological distress

Psychological distress was assessed using the BSI-18 which comprises three subscales of anxiety, depression, and somatization, as well as a global severity index (GSI) which is the sum of the three subscales. A 5-point Likert scale is utilized for each of the 18 items from 0 indicating not at all to 4 indicating always with the timespan relating to the past 7 days. Each subscale ranges from 0 to 24 with the GSI ranging from 0 to 72. For clinical case finding, raw scores were converted to gender-specific T-scores (with a mean of 50 and a standard deviation of 10) with the following case-rules applied: (1) Standard case-rule, a GSI ≥ 63 or at least two of the sub-scales with a T-score ≥ 63 [24]; (2) Zabora case-rule, a GSI T-score ≥ 57 [25]; and (3) Recklitis case-rule, if the GSI ≥ 50 [26]. The BSI-18 is a reliable instrument for the assessment of psychological distress [27] where higher scores on the subscales and the GSI indicate higher distress [28].

### Other measures

Height and weight were assessed using a stadiometer and electronic scales, respectively, with body fat percentage determined by dual-energy X-ray absorptiometry (DXA, Hologic Discovery A, Waltham, MA, USA). Muscle strength was determined for the chest press, leg press, seated row, and leg extension using the one-repetition maximum (1-RM) and reported as average strength, the 400-m walk was used as a measure of aerobic capacity, the 6-m backwards walk as a measure of dynamic balance, and the repeated chair rise to standing (five times) for lower body muscle function [29]. Testosterone and PSA were measured commercially by an accredited Australian National Association of Testing Authorities laboratory (Pathwest Diagnostics, Perth, Western Australia).

### Statistical analyses

Data were analyzed using IBM SPSS Version 24. Normality of distribution was assessed using the Kolmogorov–Smirnov test. Differences among groups at baseline were assessed using one-way analysis of variance (ANOVA) or the Kruskall–Wallis test, as appropriate, for continuous data and chi-square for categorical data. Changes over the 12-month study period were assessed using a two-way (group × time) repeated measures ANOVA. Follow-up tests were performed if the interaction or main effect for time was significant. Where appropriate the Bonferroni post-hoc procedure for multiple comparisons was used to locate the source of significant differences. Data not normally distributed were log transformed (ln) for analysis with ln (*x* + 2) used for the BSI subscales and global scale as scores included zero. To examine change in psychological distress following supervised exercise (ImpRes, baseline to 12 months; AerRes, baseline to 6 months; and DelAer, 6 months to 12 months), trend analysis was performed using linear regression and entering tertiles of anxiety, depression, somatization, and the GSI as an ordinal variable. The Mann–Whitney *U* test was used to examine magnitude of change following supervised exercise between clinical cases and non-cases according to the Zabora case rule and Recklitis case rule for anxiety, depression, somatization, and the GSI. Intention-to-treat was utilized for analyses using maximum likelihood imputation of missing values (expectation maximization). Tests were two-tailed with statistical significance set at an alpha level of 0.05.

## Results

There were no differences among groups at baseline for any demographic or clinical characteristic (Table 1). Men were aged 43–90 years with a body fat 12.9–45.1%, were mostly married, not currently employed, and non-smokers. The exercise program had the desired effect resulting in a progressive improvement in muscle strength over the 12-month period in ImpRes (*p* < 0.001) and in the first 6 months in AerRes (*p* < 0.001) which was then maintained at 12 months (Table 2). Similarly, there were progressive improvements in the 6-m backwards walk (*p* < 0.001) and chair rise ability (*p* < 0.001) over 12 months in ImpRes with the 400-m walk time (*p* = 0.005) improving by 12 months, whereas in AerRes the improvement in 400-m walk (*p* < 0.001) and chair rise (*p* < 0.001) occurred by 6 months and was then maintained during the 6-month home-based period. For DelAer, the improvements (*p* < 0.01) were mostly observed after the second 6-month period following exercise. As previously reported, there were no adverse events directly related to the exercise programs undertaken [20].

**Table 1:** Participant characteristics.

	ImpRes	AerRes	DelAer	*p* value
	(*n* = 49)	(*n* = 50)	(*n* = 36)	
Age, yr (SD)	68.7 ± 9.3	69.1 ± 9.6	69.7 ± 8.4	0.869
Height, cm (SD)	173.5 ± 5.8	173.1 ± 6.7	171.9 ± 5.4	0.467
Weight, kg (SD)	84.5 ± 11.5	85.0 ± 16.0	87.6 ± 14.4	0.565
Body fat, % (SD)	27.7 ± 4.8	27.5 ± 6.1	29.1 ± 4.5	0.350
Employed, *N* (%)	22 (38.6)	17 (31.5)	19 (38.8)	0.571
Married, *N* (%)	44 (77.2)	42 (77.8)	43 (87.8)	0.720
Current smoker, *N* (%)	3 (5.3)	3 (5.6)	3 (6.1)	0.822
Gleason score^a^	7.0 (7.0–8.3)	8.0 (7.0–9.0)	7.0 (7.0–8.3)	0.411
ADT time, months^a^	3.0 (2.0–4.5)	3.0 (2.0–4.3)	3.0 (2.0–4.0)	0.624
PSA, ng/mL^a^	0.4 (0.1–1.2)	0.2 (0.0–1.0)	0.4 (0.0–1.0)	0.599
Testosterone, pg/mL^a^	0.0 (0.0–1.3)	0.8 (0.0–1.1)	0.5 (0.0–0.9)	0.973
Other Conditions, *N* (%)				
Cardiovascular disease	3 (6.1)	3 (6.0)	2 (5.6)	0.994
Hypertension	17 (34.7)	15 (30.0)	19 (52.8)	0.085
Dyslipidemia	9 (18.4)	13 (26.0)	9 (25.0)	0.626
Diabetes	3 (6.1)	7 (14.0)	7 (19.4)	0.175

*ImpRes* impact plus resistance exercise; *AerRes* aerobic plus resistance exercise; *DelAer* delayed followed by aerobic exercise; *SD* standard deviation; *PSA* prostate specific antigen.

^a^Median and interquartile range (IQR); missing vales: employed *n* = 5, married *n* = 5, smoker *n* = 3, PSA *n* = 5, testosterone *n* = 6, Gleason score *n* = 14.

**Table 2:** Physical performance measures at baseline, 6 and 12 months.

	Baseline (0)	6 months	12 months	*P* value		
				Group × Time	Time	Comparison^a^
Average muscle strength (kg)						
ImpRes	70.5 ± 21.2	81.6 ± 22.0	86.9 ± 22.0	<0.001	<0.001	0 < 6 < 12
AerRes	71.2 ± 22.2	82.6 ± 23.3	81.1 ± 22.8			0 < 6, 12
DelAer	70.8 ± 26.1	71.5 ± 23.7	77.4 ± 27.0			0, 6 < 12
400-m walk (s)^b^						
ImpRes	274.6 ± 58.1	263.1 ± 49.1	259.4 ± 49.8	0.505	<0.001	0 > 12
AerRes	270.1 ± 52.6	255.1 ± 42.8	256.9 ± 49.2			0 > 6, 12
DelAer	277.1 ± 47.8	270.0 ± 45.5	267.9 ± 46.0			0 > 12
6-m backwards walk (s)^b^						
ImpRes	19.3 ± 8.5	16.7 ± 6.4	15.8 ± 6.8	0.173	<0.001	0 > 6 > 12
AerRes	19.0 ± 7.2	17.1 ± 6.0	16.0 ± 6.1			0 > 6 > 12
DelAer	19.0 ± 7.3	18.6 ± 8.7	18.1 ± 9.7			
Chair rise (s)^b^						
ImpRes	13.3 ± 4.3	11.9 ± 3.9	10.9 ± 2.8	0.005	<0.001	0 > 6 > 12
AerRes	12.8 ± 2.8	11.8 ± 2.6	11.6 ± 2.2			0 > 6, 12
DelAer	14.4 ± 3.9	13.3 ± 2.8	11.6 ± 1.9			0 > 6 > 12

Values are the mean ± SD.

*ImpRes* impact plus resistance exercise; *AerRes* aerobic plus resistance exercise; *DelAer* delayed followed by aerobic exercise.

^a^Within-group multiple comparisons for baseline (0), 6, and 12 months, with a Bonferroni-corrected *p* < 0.05.

^b^Statistical analysis based on log transformed data.

### Psychological distress

At baseline there were no differences among groups for the three subscales anxiety, depression, and somatization, or the GSI (*p* = 0.132–0.694). There was no change in somatization over the study period, however, there were significant interactions for depression (*p* < 0.001), anxiety (*p* = 0.002), and GSI (*p* < 0.001) (Table 3). In ImpRes, depressive symptoms and the GSI were reduced at 12 months compared to baseline and 6 months, while anxiety was also reduced at 12 months compared to baseline. For those in the AerRes group, depressive symptoms and the GSI tended to decline following supervised exercise but then increased following the non-supervised home-based period such that depressive symptoms at 12 months were greater than at baseline and 6 months and the GSI was also higher at 12 months than 6 months. A similar pattern was also evident for anxiety which tended to be reduced following supervised exercise but then increased following the non-supervised period such that anxiety symptoms were greater at 12 than 6 months in AerRes. For those in DelAer, there was no significant change in any subscale while the GSI was reduced at 12 months following exercise compared to 6 months. However, it should be noted that although scores in the three subscales and the GSI did not significantly change following the usual care period they were all higher than at baseline.

**Table 3:** Psychological distress based on BSI-18 at baseline, 6, and 12 months.

	Baseline (0)	6 months	12 months	*P* value		
				Group × Time	Time	Comparison^a^
Anxiety						
ImpRes	1.98 ± 2.56	1.27 ± 1.38	1.08 ± 1.54	0.002	0.098	0 > 12
AerRes	1.30 ± 1.97	0.91 ± 1.45	1.38 ± 1.77			6 < 12
DelAer	1.36 ± 2.46	1.41 ± 1.74	1.18 ± 1.80			
Depression						
ImpRes	1.88 ± 3.24	1.48 ± 2.65	0.78 ± 1.39	<0.001	0.623	0, 6 > 12
AerRes	1.46 ± 3.57	1.08 ± 1.76	1.70 ± 2.11			0, 6 < 12
DelAer	1.33 ± 1.71	1.45 ± 1.82	1.01 ± 1.24			
Somatization						
ImpRes	2.08 ± 2.92	1.89 ± 1.88	1.82 ± 2.00	0.148	0.241	
AerRes	1.58 ± 2.02	1.30 ± 1.54	1.46 ± 1.76			
DelAer	1.97 ± 2.38	2.34 ± 2.21	1.63 ± 1.89			
Global Severity Index						
ImpRes	5.94 ± 7.46	4.64 ± 4.73	3.67 ± 4.34	<0.001	0.074	0, 6 > 12
AerRes	4.34 ± 6.46	3.29 ± 4.19	4.49 ± 5.05			6 < 12
DelAer	4.67 ± 5.50	5.25 ± 4.22	3.78 ± 3.94			6 > 12

Values are the mean ± SD.

*ImpRes* impact plus resistance exercise, *AerRes* aerobic plus resistance exercise, *DelAer* delayed followed by aerobic exercise.

^a^Within-group multiple comparisons for baseline (0), 6, and 12 months, with a Bonferroni-corrected *p* < 0.05; Statistical analysis based on log transformed data.

**Fig. 1:**
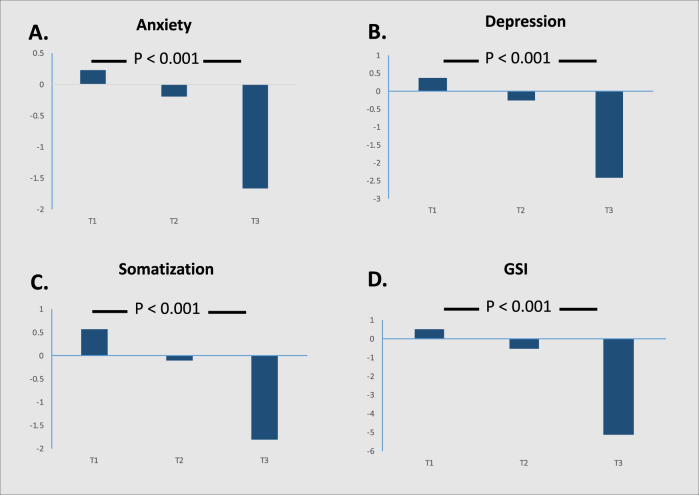
Change in anxiety (**A**), depression (**B**), somatization (**C**), and the global severity index (GSI, **D**) following supervised exercise (ImpRes from baseline to 12 months, AerRes from baseline to 6 months, and DelAer from 6 months to 12 months). Tertile 1 (T1) lowest symptoms to tertile 3 (T3) highest symptoms. *P*-value for trend analysis.

When anxiety, depression, somatization, and the GSI were examined by tertiles, there was a significant trend (*p*_trend_ < 0.001) for those in the highest tertile of each subscale and the GSI prior to the initiation of exercise (i.e., higher distress) to derive the most benefit following supervised exercise, that is, have a reduction in psychological distress (Fig. 1).

When T-scores were calculated, the mean scores at baseline were within the normal range (anxiety mean of 46.0 and SD = 7.4, depression mean of 46.5 and SD = 7.6, somatization mean of 49.2 and SD = 7.7, and GSI mean of 47.5 and SD = 8.2). The number of cases of clinical distress based on the Standard, Zabora and Recklitis case rules at baseline, 6 and 12 months, for ImpRes, AerRes and DelAer are shown in Table 4. With the different criteria the number of cases varied with most cases identified using the Recklitis case-rule which has the lowest GSI cut-off value. For all three case rules, the number of cases were less at 6 months following exercise in ImpRes and AerRes with further reduced cases in ImpRes at 12 months when the Zabora and Recklitis case rules were applied. Conversely, the number of cases were higher at 12 months in AerRes following the non-supervised 6-month exercise period, while the number of cases of distress were reduced in DelAer following supervised training from 6 to 12 months. Based on the Zabora and Recklitis case finding rules, those classed as clinically distressed responded significantly better to supervised exercise than non-cases (Supplementary Table).

**Table 4:** Case finding for clinical distress at baseline, 6 and 12 months.

	Baseline		6 months		12 months	
	Case	Non-case	Case	Non-case	Case	Non-case
Standard Case Rule^a^						
ImpRes	2	47	1	48	1	48
AerRes	3	47	1	49	2	48
DelAer	1	35	0	36	0	36
Zabora Case Rule^b^						
ImpRes	7	42	4	45	3	46
AerRes	6	44	5	45	7	43
DelAer	4	32	5	31	2	34
Recklitis Case Rule^c^						
ImpRes	23	26	20	29	17	32
AerRes	14	36	10	40	15	35
DelAer	15	21	16	20	13	23

*ImpRes* impact plus resistance exercise; *AerRes* aerobic plus resistance exercise; *DelAer* delayed followed by aerobic exercise.

^a^Standard BSI-18 case rule, a global severity index (GSI) ≥ 63 or at least 2 of the sub-scales with a T-score ≥ 63.

^b^Zabora case rule, a GSI T-score ≥ 57.

^c^Recklitis case rule, a GSI ≥ 50.

## Discussion

To our knowledge, this is the first RCT to examine the long-term effects of different exercise modalities on psychological distress in men with PCa undergoing ADT. There were three important findings: (1) various modes of supervised exercise were effective in reducing psychological distress in men with PCa on ADT; (2) men with the highest level of psychological distress improved the most as a result of exercise; and (3) all exercise modes led to improvements in objectively measured physical function.

Psychological distress is common in men with PCa across treatment modalities and stages of disease. The prevalence of depression and anxiety in men with PCa across treatment trajectories has been estimated to be between 15 and 27% [12]. In our sample, the prevalence of clinical distress at baseline ranged between 4 and 38% as this variation was dependent on the case rule employed (4.4% Standard; 12.6% Zabora; and 38.5% Recklitis). Exercise has been proposed to have a significant antidepressant effect in people with depression [15]. However, previous systematic reviews reported insufficient data available or only initial positive trends for the effect of exercise on anxiety and depression in this patient group [18, 19]. Here, we expand on these initial findings by reporting the long-term effect of different exercise modalities on psychological distress in men with PCa undertaking ADT. The results indicate that various modes of supervised exercise are effective at reducing elements of psychological distress in men with PCa receiving ADT. Interestingly, in two of the exercise groups (ImpRes and AerRes), the exercise interventions included some form of resistance-based exercise which has been shown to be an important exercise mode to reduce depressive symptoms in adults [17].

Importantly, the positive improvements in elements of psychological distress in this study were largely a result of the supervised portion of the exercise program in each group. These results are consistent with those of a meta-analysis demonstrating that the effects of exercise on depressive symptoms were larger with programs supervised or partially supervised [30]. Thus, elements of a supervised exercise program such as social interaction with peers and professionals, learning new skills and receiving positive feedback may contribute to improvements in symptoms of distress [30, 31]. From 6–12 months the AerRes patients continued to perform resistance training, but at home with elastic bands and the likely reduced intensity and volume of resistance exercise may have also contributed to the initial benefits of clinic-based exercise being diluted. We have recently proposed strategies to improve the long-term efficacy and adherence in non-clinic settings using digital health technology such as the use of wearable sensors, utilizing digital exercise platforms for prescription, delivery, and instruction, and video chat with a qualified exercise professional to monitor and support the patient [32].

We also observed that men with the highest level of psychological distress improved the most as a result of exercise. Distress screening for men with PCa has been well validated [33] and contemporary models of psychological care in cancer propose a stepped approach where the depth of intervention is matched to the extent of distress [34]. As we proposed for patients with higher fatigue [20], and in this case for distress, screening patients on ADT for such symptoms and directing tailored and supervised exercise interventions to those with clinical symptoms should be part of PCa treatment care strategies. Further, we would hypothesize that interventions that integrate tailored exercise programs with evidence-based psychological interventions [13] may well provide the optimal platform for survivorship care for this patient group [35].

As expected, the intervention also improved physical function and these results are consistent with previous work undertaken by our group and others [29, 36]. These changes are clinically important as ADT is associated with reduced muscle strength, functional performance, balance, and musculoskeletal health increasing the risk of falls and fractures in these patients [37]. Interestingly, all exercise modes led to similar improvements in physical function suggesting that patients on ADT can benefit from an array of exercise programs when supervised and at appropriate intensity and dosage.

Of interest, we recently reported in a comprehensive meta-analysis and meta-regression that low volume resistance-based exercise undertaken at a moderate-to-high intensity led to improvements in fatigue and quality of life, and also mitigated depression and anxiety symptoms in men with prostate cancer [38]. The results from our current study are in agreement with this but also suggest that low volume aerobic exercise may be beneficial in improving psychological distress.

Our study has several strengths and limitations worthy of comment. This is the largest RCT to date examining the effect of different supervised exercise modes including resistance, impact loading and aerobic training on psychological distress in men with PCa on ADT. Psychological distress was assessed using the BSI-18 which includes subscales of anxiety, depression, somatization, and GSI and is a reliable instrument for the assessment of psychological distress. Nevertheless, there are some limitations that warrant attention. Although positive effects of exercise on psychological distress were found, we did not specifically target patients who were diagnosed or distressed using specific cutoffs. Thus, an investigation of the impact of different exercise modalities on symptoms in those clinically distressed or depressed to gain further understanding of the interaction between exercise and personal characteristics is warranted. However, our results do indicate that those with higher scores for anxiety, depression, and somatization, as well as the GSI, and those classed as clinically distressed based on the case-finding rules employed in the study, responded better to exercise. In addition, although patients in the AerRes group were encouraged to accumulate further activity, the volume of additional activity outside of the supervised sessions was not recorded. Lastly, the men in this study were primarily in their first year of ADT. Thus, these results may not be generalizable to men on different treatment regimens or of a more advanced disease stage.

In summary, various exercise modes when supervised are effective in reducing psychological distress in men with PCa on ADT. Moreover, those with the highest level of psychological distress improved the most. Supervised exercise can be prescribed to improve psychological health in PCa patients on ADT. As a practical clinical recommendation, the American College of Sports Medicine has proposed a physician assessment and referral strategy to exercise specialists that can be used as a model to facilitate exercise supervision in the setting of oncology [39]. These strategies are relevant to men with PCa and should be used by their treating urologists/oncologists to improve exercise participation and improve psychological distress.

## Supplementary information

Supplemental material
