# Supplementary material for: Psychological distress in men with prostate cancer undertaking androgen deprivation therapy: modifying effects of exercise from a year-long randomized controlled trial
Source: Prostate Cancer Prostatic Dis. 2021 Feb 8;24(3):758–66. doi: 10.1038/s41391-021-00327-2 (PMC8384619; doi:10.1038/s41391-021-00327-2)
Supplement: Supplementary file 1 — Supplemental material [file 41391_2021_327_MOESM1_ESM.docx]

Supplementary Table. Change in anxiety, depression, somatization, and the global severity index following supervised exercise based on clinical case finding prior to exercise.

Cases Non-cases P-value

Zabora Case Rule^a^

Anxiety -3.00 (-4.00, -0.93) 0.00 (-0.86, 0.00) <0.001

Depression -2.54 (-8.00, 0.00) 0.00 (-0.64, 0.04) 0.004

Somatization -1.76 (-3.50, -0.27) 0.00 (-0.95, 0.56) 0.003

GSI -7.00 (-14.00, -3.00) -0.40 (-2.00, 0.11) <0.001

Recklitis Case Rule^b^

Anxiety -1.00 (-2.00, 0.00) 0.00 (-0.35, 0.04) <0.001

Depression -0.99 (-2.88, 0.00) 0.00 (0.00, 0.14) <0.001

Somatization -0.87 (-2.11, 0.26) 0.00 (-0.42, 0.53) 0.021

GSI -3.14 (-5.00, -0.53) 0.00 (-1.00, 1.00) <0.001

^a^Zabora case rule, a GSI T-score ≥ 57; ^b^Recklitis case rule, a GSI ≥ 50. GSI = global severity index. Values are the median and interquartile range (IQR).
